# Disclosing 3' UTR *cis*-elements and putative partners involved in gene expression regulation in *Leishmania* spp.

**DOI:** 10.1371/journal.pone.0183401

**Published:** 2017-08-31

**Authors:** Monica Cristina Terrão, Elton José Rosas de Vasconcelos, Tânia Aquino Defina, Peter J. Myler, Angela Kaysel Cruz

**Affiliations:** 1 Department of Cell and Molecular Biology, Ribeirao Preto School of Medicine, University of São Paulo, Ribeirão Preto, Brazil; 2 Center for Infectious Disease Research, 307 Westlake Avenue, Seattle, Washington, United States of America; 3 Department of Global Health, University of Washington, Seattle, Washington, United States of America; 4 Department of Biomedical Informatics and Medical Education, University of Washington, Seattle, Washington, United States of America; Meharry Medical College, UNITED STATES

## Abstract

To identify putative *cis*-elements involved in gene expression regulation in *Leishmania*, we previously conducted an *in silico* investigation to find conserved intercoding sequences (CICS) in the genomes of *L*. *major*, *L*. *infantum*, and *L*. *braziliensis*. Here, the CICS databank was explored to search for sequences that were present in the untranslated regions (UTRs) of groups of genes showing similar expression profiles during *in vitro* differentiation. Using a selectable marker as a reporter gene, flanked by either an intact 3' UTR or a UTR lacking the conserved element, the regulatory role of a CICS was confirmed. We observed that the pattern of modulation of the mRNA levels was altered in the absence of the CICS. We also identified putative CICS RNA-binding proteins. This study suggests that the publicly available CICS database is a useful tool for identifying regulatory *cis*-elements for *Leishmania* genes and suggests the existence of post-transcriptional regulons in *Leishmania*.

## 1 Introduction

The protozoan parasite *Leishmania* is the causative agent of leishmaniasis, a spectrum of diseases that range in severity from spontaneously healing cutaneous lesions to potentially fatal visceral disease [1]. In addition to its medical importance, this ancient eukaryote is used as a model organism for studying genetic organization and regulation of gene expression. In this parasite, as in other kinetoplastids and distinct from most eukaryotes, processing of mRNAs occurs by *trans*-splicing, transcription is polycistronic and constitutive, and gene expression is controlled mostly post-transcriptionally [2].

In general, the organisms adapt to changes in their environment by modulating their gene expression profile. In heteroxenous parasites such as *Leishmania* and other trypanosomatids, adaptation to diverse and hostile environments requires an immediate response involving morphological and physiological changes driven by modifications in gene expression. While transcription initiation is the primary point of gene expression control in most eukaryotes, trypanosomatid gene expression lacks any processes regulated by RNA polymerase II [2]. In fact, the available data show that genes in these parasites are transcribed and processed continuously, and gene expression is regulated by selective transport to the cytoplasm, mRNA stability or translation initiation [3].

A considerable amount of information on the genomes, transcriptomes, proteomes and metabolomes of trypanosomatids is now available [4]. Accessible data expedites investigations regarding the biology of these parasites and represents a major tool for studies of the regulation of gene expression, an area with much remaining to be understood [5].

Many studies on the mechanisms of gene expression control in trypanosomatids have demonstrated the role of *cis*-elements present in the 3' untranslated region (3' UTR) of transcripts, which in association with RNA-binding proteins (RBP) modulate gene expression. These *cis*- and *trans*-acting elements are involved in controlling either transcript stability or translation efficiency [2, 6]. Well-characterized regulatory *cis*-elements include the AU-rich elements (ARE), which bind to RBPs containing RNA recognition motifs (RRM) known as RRM-type RBPs to modify the stability (half-life) of RNAs [7]. In *Leishmania*, in addition to the AREs, a large family of extinct retroposons termed the SIDERs (Short Interspersed DEgenerated Retroposons) is also involved in post-transcriptional regulation of gene expression. SIDERs are widely distributed within the 3' UTRs of unrelated transcripts [8–10]. Thus, investigation of the *cis*-elements and their associated proteins is important to improve the understanding of how gene expression is regulated in different environments by this parasite.

To identify regulatory *cis*-elements, we developed a computational pipeline that isolated conserved sequences present in the genomes of *L*. *braziliensis*, *L*. *infantum* and *L*. *major* [11]. The pipeline was designed to exclude annotated CDSs in order to best compare the remaining intercoding regions. The 9,225 conserved intercoding sequences (CICS) found in the three genomes, named LeishCICS, are available as supplementary material elsewhere [11].

To assess the putative functional role of CICS and characterize them as regulatory elements, in this study four CICS were investigated and their putative RBPs partners were identified. CICS 1722 and 4405 were investigated as *cis*- regulatory elements, and the role of CICS1722 in the regulation of gene expression in *Leishmania* was experimentally confirmed.

## 2 Materials and methods

### 2.1 Cell line, culture conditions, *in vitro* differentiation and transfection

Promastigotes of *Leishmania donovani* LdBOB (MHOM/SD/62/1S-CL2D) [12] were grown at 26°C in M199 medium (Gibco BRL), supplemented as described by Kapler [13]. Differentiation of promastigotes to amastigotes in axenic culture was conducted as described by Barak [14], S1 Fig.

Cells grown to late log phase were transfected by electroporation (500 μF and voltage of 2.25 kV/cm), and stable transfectants were selected in solid medium with 2X DL_50_ of G418, as previously described by Kapler [13]. After selection, transfectants were maintained in the absence of drug pressure.

To measure mRNA stability, transcription initiation and spliced leader RNA methylation were inhibited by treating cells with 1 μg/ml of Sinefungin for 5 min at room temperature, followed by the addition of Actinomycin D (10 μg/ml). Samples were collected at 0, 30, 60 and 120 min after adding Actinomycin D.

### 2.2 Constructs and oligonucleotides

To remove the CICS from its genomic context, we ordered DNA constructs from GenScript USA Inc. The constructs bore the reporter gene/selectable marker NEO (Neomycin Phosphotransferase) flanked by 700-bp fragments of the 5' and 3' regions present in the original locus. Sites for the restriction enzyme *Nde*I were added, flanking the CICS in the 3' UTR, enabling its removal in a single cloning step (S2 Fig). Details of all oligonucleotides used in this study are provided in the Table 1.

**Table 1:** Oligonucleotides used in this study.

Primer	Sequence 5' → 3'
NEO-RT-F	AGACAATCGGCTGCTCTGAT
NEO-RT-R	CTCGTCCTGCAGTTCATTCA
Actin-RT-F	TGGCACCATACCTTCTACAACGAG
Actin-RT-R	CGTCATCTTCTCACGGTTCTGC
5UTR-Lin07.0150-F	ATCAGCTACAACCCGTGTCC
5UTR-Lin31.1630-F	CTACCTTCTTGACCTTCGCG
NEO::DHFR-F	TCGCCTTCTTGACGAGTTCT
DHFR::NEO-R	TAGCCGAATAGCCTCTCCAC
RNA-Control	UCCUGCUUCAACAGUGCUUGGACGGAAC-Biotin
NEO-end-F	GCATCGCCTTCTATCGCCTT
3UTR-07.0150-R	CGGCTCATTCTAGCAGCTCA
3UTR-31.1630-R	GCAAACGTGTCCACTGTCGA

### 2.3 Nucleic acid isolation

Total cellular RNA was extracted from the *Leishmania* cells using TRIzol^®^. Quality and integrity of total RNA were assessed on 1% formaldehyde-agarose gels. Intact genomic DNA was prepared in agarose plugs as described by Coburn [15].

### 2.4 Protein extraction and quantification

To search for CICS-interacting proteins, total protein extract from *L*. *donovani* BOB was fractioned into nuclear and cytoplasmic extracts [16, 17]. Proteins were quantified by the Bradford method [18].

### 2.5 RNA pull-down

For the pull-down reaction, biotinylated RNA oligonucleotides synthesized by Integrated DNA Technologies (USA) were used. The biotinylated RNA (100 pmol) was immobilized in a streptavidin column (Dynabeads M-280 Streptavidin—Invitrogen, USA) in BW buffer (5 mM Tris-HCl pH 7.5, 0.5 mM EDTA, 1 M NaCl) to a final volume of 100 μL for 30 min at room temperature. The beads were washed 3 times with BW buffer, and resuspended in protein extract buffer. Non-specific interactions were removed by incubation of the protein extract with the beads (1 μl beads / 5 μg proteins) for 2 hours at 4°C on a rotating platform. The protein extract (200 μg) was incubated with the biotinylated RNA linked to the beads for 2 hours at 4°C on a rotating platform. To eliminate non-interacting proteins, the reaction mixture was washed 3 times with 100 μl of protein extract buffer containing 0.1% NP-40 [16, 17]. Bound proteins were eluted by boiling for 5 min with a 20 μl SDS sample buffer (100 mM Tris-Cl pH 6.8, 4% (w/v) SDS, 0.2% (w/v) bromophenol blue, 20% (v/v) glycerol, 200 mM DTT) and subjected to SDS-PAGE. The proteins identified as interacting with the RNA-free beads and those interacting with a control 28-mer 3' biotinylated RNA sequence absent from the *Leishmania* genome (Table 1) were used as negative controls and subtracted from the group of proteins that bound to the CICS. The cutoff for including a protein as a CICS-binding protein was the detection of 2 unique peptides in the MS profile.

### 2.6 Southern blotting

Standard protocols were used for Southern blotting and hybridization analyses. The NEO probe consisted of a 210-bp fragment of the protein coding sequence from the pX63NEO vector [19], digested with the restriction enzyme *Sap*I and labeled using the random primer method [20].

### 2.7 Real-time PCR and relative quantification

RT-qPCR was performed using an ABI 7500 Sequence Detection System (Applied Biosystems, USA) in the presence of SYBR Green. The optimization of the RT-qPCR was done according to the manufacturer’s instructions (Applied Biosystems User Bulletin 2, applied to the SYBR-Green I core reagent protocol). Reactions were conducted in technical and biological triplicates using SYBR Green (SYBR Green PCR Master Mix, Applied Biosystems, USA). To quantify NEO transcript levels, the primers NEO-RT-F and NEO-RT-R were used (Table 1). As internal controls, the β-actin (for comparison of the reporter gene in the presence and absence of the CICS) and 18S rRNA (for RNA decay experiments) genes were used. To evaluate target amplification efficiency, a standard curve was generated using 10-fold serial dilutions of cDNA. The relative expression was analyzed by the 2^-ΔΔCT^ method.

## 3 Results

### 3.1 Selection of conserved intercoding sequences (CICS)

**Fig 1:**
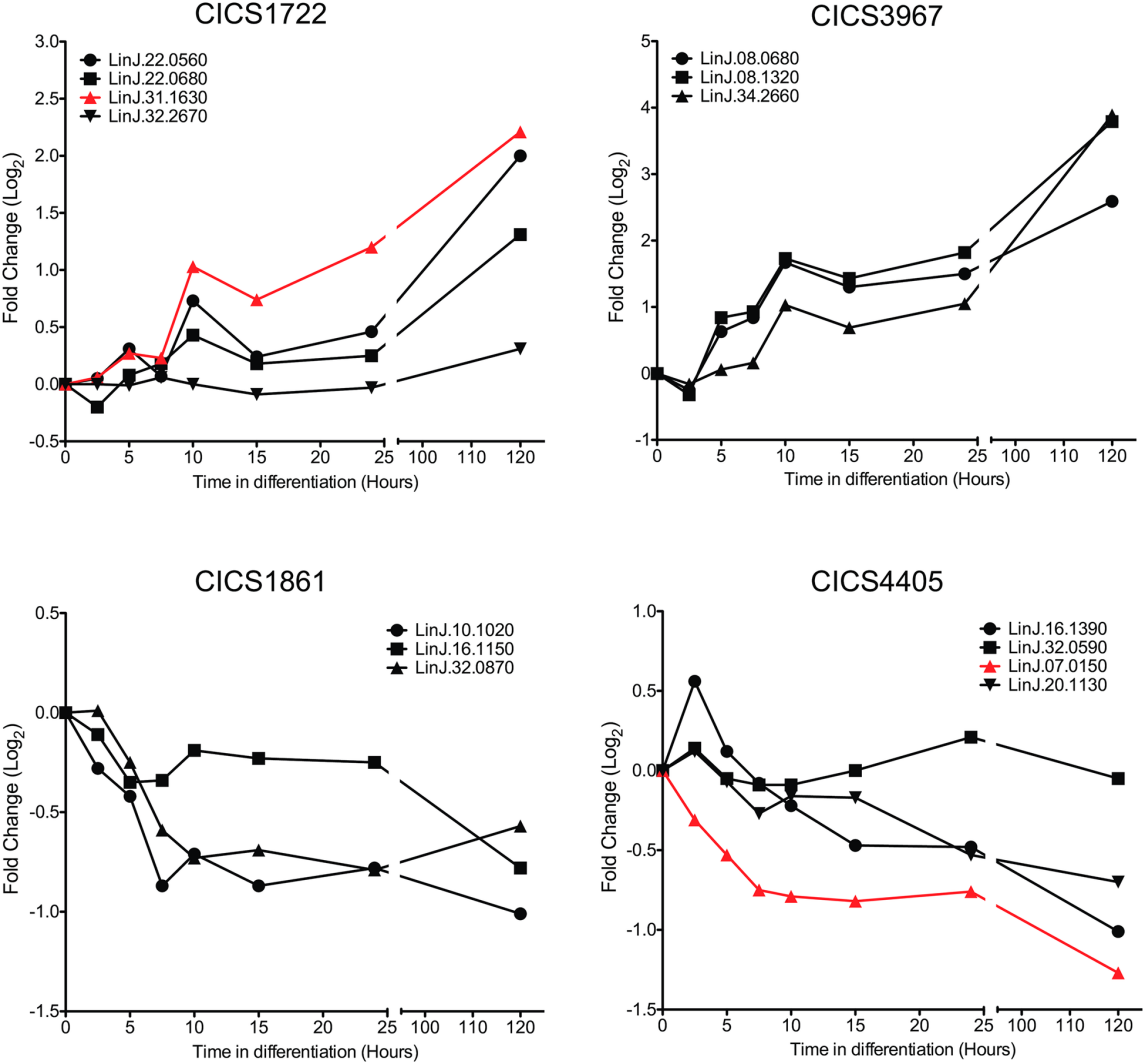
Transcript modulation pattern during differentiation. Comparison of the transcript levels of genes bearing the same CICS in their UTRs using microarray data [21]. Lahav et. al. measured transcript levels at eight time points (0, 2.5, 5, 7.5, 10, 15, 24 and 120 hours) of differentiation from promastigote to amastigote stages by microarray [21]. We collated four groups of co-regulated genes sharing the same CICS. In each group, the expression change of the gene selected for replacement by the NEO marker is depicted in red. The time points 0 hours and 120 hours are respectively equivalent to the metacyclic promastigote and amastigote stages outlined in S1 Fig.

We previously generated the LeishCICS databank [11] containing 9,225 CICS common to the genomes of three different *Leishmania* species: *Leishmania braziliensis*, *Leishmania infantum* and *Leishmania major*. We detected CICS present in the UTR of a single gene in the three species of *Leishmania*, and CICS that were common to UTRs of more than one gene, functionally related or not. We hypothesized that different genes bearing the same CICS might be part of a post-transcriptional regulon. To test this hypothesis, *L*. *donovani* transcripts bearing the same CICS and sharing similar patterns of modulation of expression during differentiation from promastigote to amastigote stages were selected. Selection of the transcripts with these features was performed using a microarray databank produced by Lahav and co-workers [4, 21]. Lahav kindly made available the information on the expression levels of *L*. *donovani* genes throughout differentiation; these data include only those genes with calculated Pearson’s correlation coefficients (PCC) between replicates above the threshold established by the authors of the microarray analysis. Those genes sharing the same CICS and presenting similar patterns of expression with a PCC close to 1 were selected as potential post-transcriptional regulons. Those “regulons” presenting the highest PCCs (CICS1722, 1861, 3967 and 4405) were selected (Table 2 and Fig 1). The location of the CICS within the 3' UTR and the sequence conservation compared to the reference for each of the genes grouped as part of the putative regulon was evaluated by BlastN^®^ analysis (S3 Fig).

**Table 2:** Genes sharing CICS.

CICS1722—GGACCCTGAGATGCCACACGCTGAGGTG—PCC 0.6465291	
**Gene ID**	**Product Description**
LinJ.22.0560	3'a2rel-related protein
LinJ.22.0680	3'a2rel-related protein
LinJ.31.1630	hypothetical protein, unknown function
LinJ.32.2670	hypothetical protein, conserved
CICS1861—TGCAGGCAGAGCACAGGGTCTCA—PCC 0.9055025	
**Gene ID**	**Product Description**
LinJ.08.0680	amastin-like protein
LinJ.08.0690	amastin-like protein
LinJ.08.0700	amastin-like protein
LinJ.08.0710	amastin-like protein
LinJ.08.0720	amastin-like protein
LinJ.08.1320	amastin-like protein
LinJ.08.1330	amastin-like protein
LinJ.34.2660	amastin-like surface protein, putative
CICS3967—GCACGCACACGCACATGCACACA—PCC 0.7041785	
**Gene ID**	**Product Description**
LinJ.29.0680	phosphate transporter, putative
LinJ.07.0870	6-phosphofructo-2-kinase-like protein
LinJ.10.1020	hypothetical protein, conserved
LinJ.16.1150	hypothetical protein, conserved
LinJ.24.0680	protein kinase, putative
LinJ.32.0870	CYC2-like cyclin, putative (CYC2)
CICS4405—GTGTGCGCGTGCGTGTGTGT—PCC 0.6059471	
**Gene ID**	**Product Description**
LinJ.16.1390	cytochrome c, putative
LinJ.31.2890	ADP-ribosylation factor, putative
LinJ.32.0590	hypothetical protein, conserved
LinJ.07.0150	acyl-CoA dehydrogenase, mitochondrial precursor, putative
LinJ.13.0470	hypothetical protein, conserved
LinJ.20.1130	hypothetical protein, conserved

PCC: Pearson’s correlation coefficient calculated for transcript levels of genes bearing the same CICS during the differentiation process, using data from Lahav *et al*. [21].

### 3.2 Confirming a functional role for CICS in gene expression regulation

To demonstrate the involvement of CICS in the regulation of gene expression, the effects of their presence on the transcript level of a reporter gene placed in the primary genomic locus were evaluated. Two of the four CICS with opposite patterns of modulation were selected (CICS1722 and 4405). Neomycin phosphotransferase (NEO) was used as a reporter gene, and constructs bearing NEO flanked by the upstream and downstream regions of the original gene were generated. Two versions of the construct for each tested CICS were designed: one keeping the entire CICS embedded in its surrounding sequences, and the other with the CICS removed from the downstream region (S2A and S2B Fig). These constructs were transfected as linear fragments to replace, by homologous recombination, one of the alleles of the primary locus with the reporter gene. Since the CICS are present in several genes, the gene presenting the most pronounced modulation profile during *in vitro* differentiation was used in the functional tests (Fig 1). The *Leishmania donovani* BOB strain was chosen because its differentiation *in vitro* is stable and highly reproducible [12].

To study CICS1722, the gene LinJ.31.1630, a conserved putative dynein heavy chain, was selected. LinJ.07.0150, a putative protein kinase-encoding gene, was selected to investigate the role of CICS4405. For each gene, constructs were designed for the replacement of one endogenous allele by the selectable marker/reporter gene flanked by sequences identical to the endogenous locus or by a similar sequence lacking the CICS (S2A and S2B Fig). The replacement of the endogenous CDS by the NEO gene, with or without the CICS, was confirmed in the *L*. *donovani* BOB transfectants by PCR (S2C and S2E Fig), Southern blotting (S2D and S2F Fig) and sequencing (S2G and S2H Fig). Importantly, after confirmation of the correct integration of the reporter gene, all transfectants were maintained in M199 with no drug selection.

**Fig 2:**
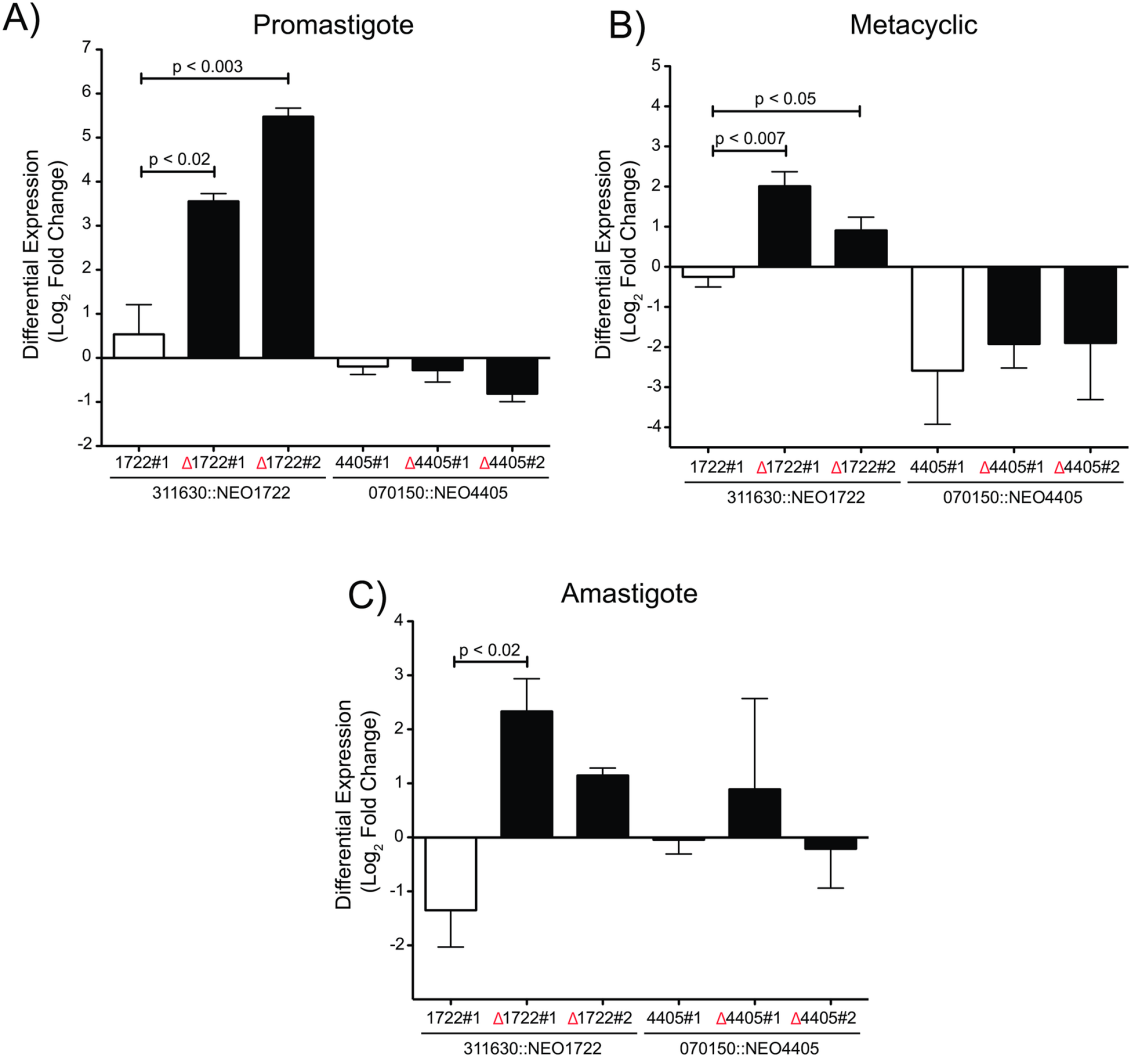
Transcript levels of the reporter gene (NEO) in the presence and absence of each CICS. The transcript levels of NEO were measured by RT-qPCR in *Leishmania* transfectants in the three stages: **(A)** procyclic promastigote, **(B)** metacyclic promastigote and **(C)** axenic amastigote. The levels of NEO expression were compared between the transfectants in the presence (white bars, clones: 311630::NEO1722#1–070150::NEO4405#1) and absence of the CICS (black bars, clones: 311630::NEOΔ1722#1 and #2–070150::NEOΔ4405#1 and #2). P-values (p) were calculated using a two-way ANOVA F-test.

To analyze the role of the CICS in the control of gene expression, reporter transcript levels were measured in promastigotes (mid-log phase), metacyclic-enriched culture (stationary phase) and axenic amastigotes (Fig 2 and S1 Fig). The comparative analysis of NEO transcript levels between the various transfectants in each of the stages was conducted by RT-qPCR. The 311630::NEO1722 transfectants revealed that, in the absence of CICS1722 (311630::NEOΔ1722), reporter transcript levels were higher in the procyclic promastigote and metacyclic-enriched cultures when compared to control transfectant, in which NEO was accompanied by the intact 3' downstream region (Fig 2A and 2B). In amastigotes, only one of the transfectants lacking CICS1722 (311630::NEOΔ1722 #1) presented significantly increased levels of the reporter transcript in the absence of this CICS (Fig 2C). In contrast, no significant differences in NEO transcript levels were observed when comparing the transfectants in the presence and absence of CICS4405 under the same conditions (Fig 2).

### 3.3 CICS1722 may target mRNA for degradation

**Fig 3:**
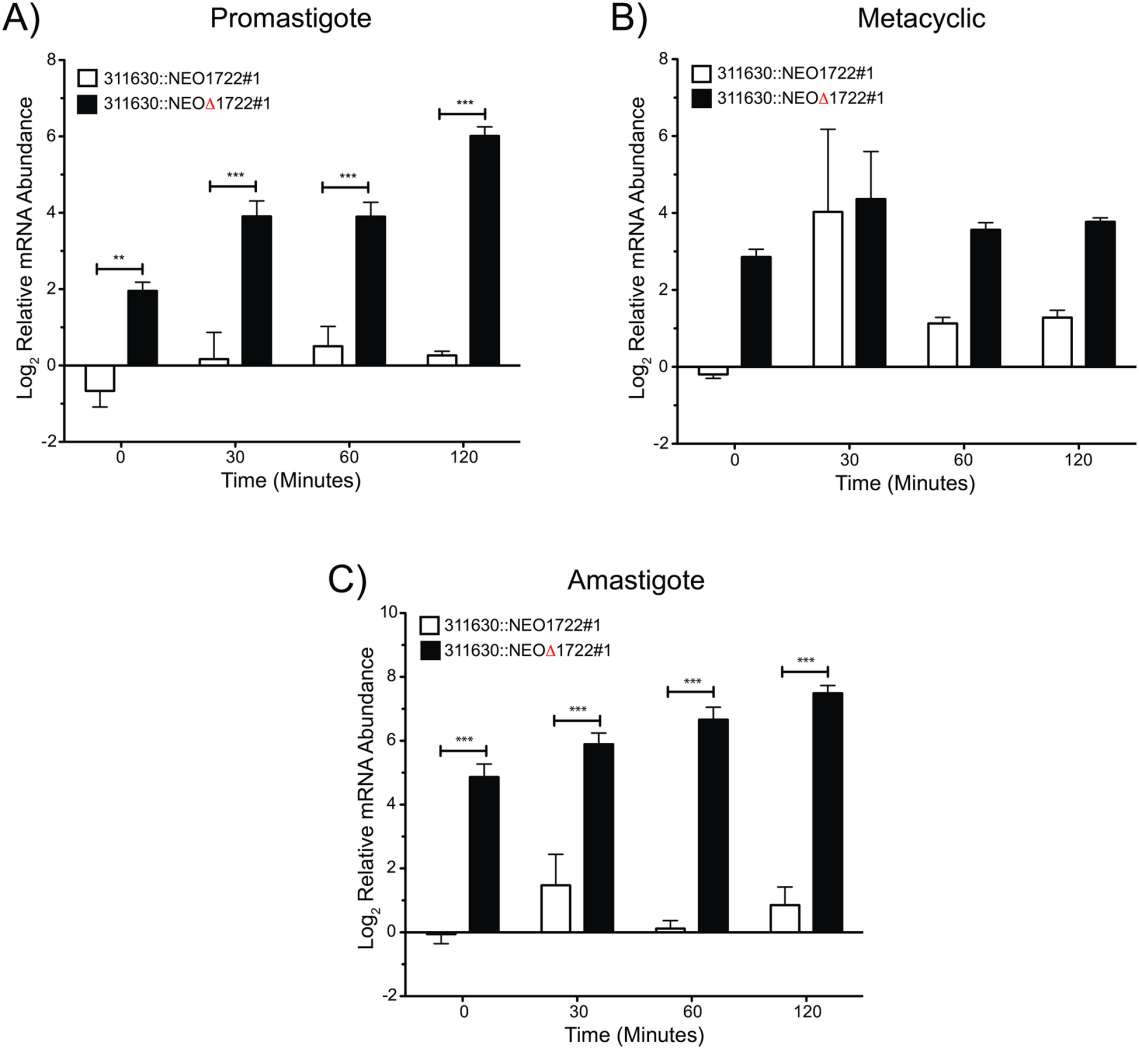
Effects of CICS on NEO mRNA stability. The transcript levels of the reporter gene were measured by RT-qPCR in the transfectants after RNA transcription inhibition by Sinefungin/Actinomycin D treatment. The levels of the NEO transcript, accompanied or not by the CICS, were compared between the transfectants. NEO inserted with CICS (white bars) and without CICS (black bars) at time points 0, 30, 60 and 120 min after treatment with transcriptional inhibitors. P-values (p) were calculated using a two-way ANOVA F-test (***, p < 0.001 and **, p < 0.01).

To identify the regulatory pathway involving CICS1722 as a *cis*-element, mRNA stability was determined in the presence and absence of CICS. For this purpose, the same transfectants were treated with Sinefungin and Actinomycin D to halt SLRNA methylation and inhibit the initiation of transcription, respectively. NEO transcript stability was determined in the treated cells by RT-qPCR; ribosomal 18S RNA was used as an internal control (Fig 3).

We demonstrated that the increase of the NEO transcript observed in the 311630::NEOΔ1722 transfectants (Fig 2) is a consequence of increased transcript stability in these conditions (Fig 3). When SLRNA methylation and initiation of transcription are inhibited by these drugs, the NEO transcript is present at significantly higher levels in the 311630::NEOΔ1722 transfectant than in the 311630::NEO1722 transfectant. The relative mRNA abundance is 4- to 6-fold higher in 311630::NEOΔ1722 promastigotes and amastigotes than in control cells (311630::NEO1722) (Fig 3A and 3C). In promastigotes and amastigotes, the NEO transcript level seems to be increasing over time in 311630::NEOΔ1722 transfectants, but this apparent accumulation of transcript over time is a consequence of the decrease in the 18S rRNA used for normalization. In the metacyclic-enriched culture, the differences between NEO transcript levels in 311630::NEO1722 and 311630::NEOΔ1722 transfectants are smaller (Fig 3B). After three experiments, these differences and a higher dispersion of values were maintained. We speculate that the variability of the mRNA reporter levels in the metacyclic stage was due to other stage-specific regulatory elements. Nevertheless, we cannot exclude the heterogeneity of cell types present in stationary cultures (the abundance of metacyclic cells, intermediate procyclic-metacyclic cells and old/sick cells are variable) as the source of the observed dispersion and the minor difference in the metacyclic-enriched culture. These results indicate that CICS1722 is a *cis*-element that acts negatively on the stability of the transcript, and in its absence the transcript gains stability.

### 3.4 Interaction of CICS with proteins

Regulatory *cis*-elements are binding sites for *trans*-acting factors (e.g., translational machinery, RBPs and ncRNAs), and the combination of these elements with different *trans*-acting factors composes the machinery responsible for one of the post-transcriptional mechanisms of gene expression control. Identification of RBPs interacting with the CICS is an important step toward elucidating the regulatory machinery. To retrieve the putative protein partners of the investigated CICS, RNA sequences of CICS1722, 4405, 1861 and 3967 were used in pull-down experiments. Nuclear extracts of mid-log-phase promastigotes and the associated binding proteins were resolved using SDS-PAGE (S4 Fig). Protein bands were detected in all pull-down experiments done with each CICS, and no bands were observed in the negative controls (S4 Fig). Twenty-four bands extracted from the gel were submitted to mass spectrometry analysis, and 33% of them were identified (Table 3). The low percentage of identified proteins may be due to low amounts of proteins in gel bands or to problems associated with sample processing. The LiAlba3 protein (LinJ.34.2410) [22] was co-purified with CICS1722 and 1861, while the RBP LinJ.35.2240 was co-purified with CICS1722, 1861 and 3967.

**Table 3:** Proteins retrieved in the pull-down experiments with indicated CICS.

Gel Fragment	Gene ID	Product Description	Gene Ontology / Molecular Function
1722a	LinJ.35.2240	RNA-binding protein, putative	nucleic acid binding
1722b	LinJ.34.2410	hypothetical protein, conserved	nucleic acid binding
1722c	LinJ.04.0750	60S ribosomal protein L10, putative	structural constituent of ribosome
1722d	LinJ.34.2410	hypothetical protein, conserved	nucleic acid binding
1861a	LinJ.32.0410	ATP-dependent RNA helicase, putative	ATP binding, ATP-dependent helicase activity, helicase activity, nucleic acid binding
1861c	LinJ.35.2240	RNA-binding protein, putative	nucleic acid binding
1861d	LinJ.34.2410	hypothetical protein, conserved	nucleic acid binding
3967c	LinJ.34.2410	hypothetical protein, conserved	nucleic acid binding

**Fig 4:**
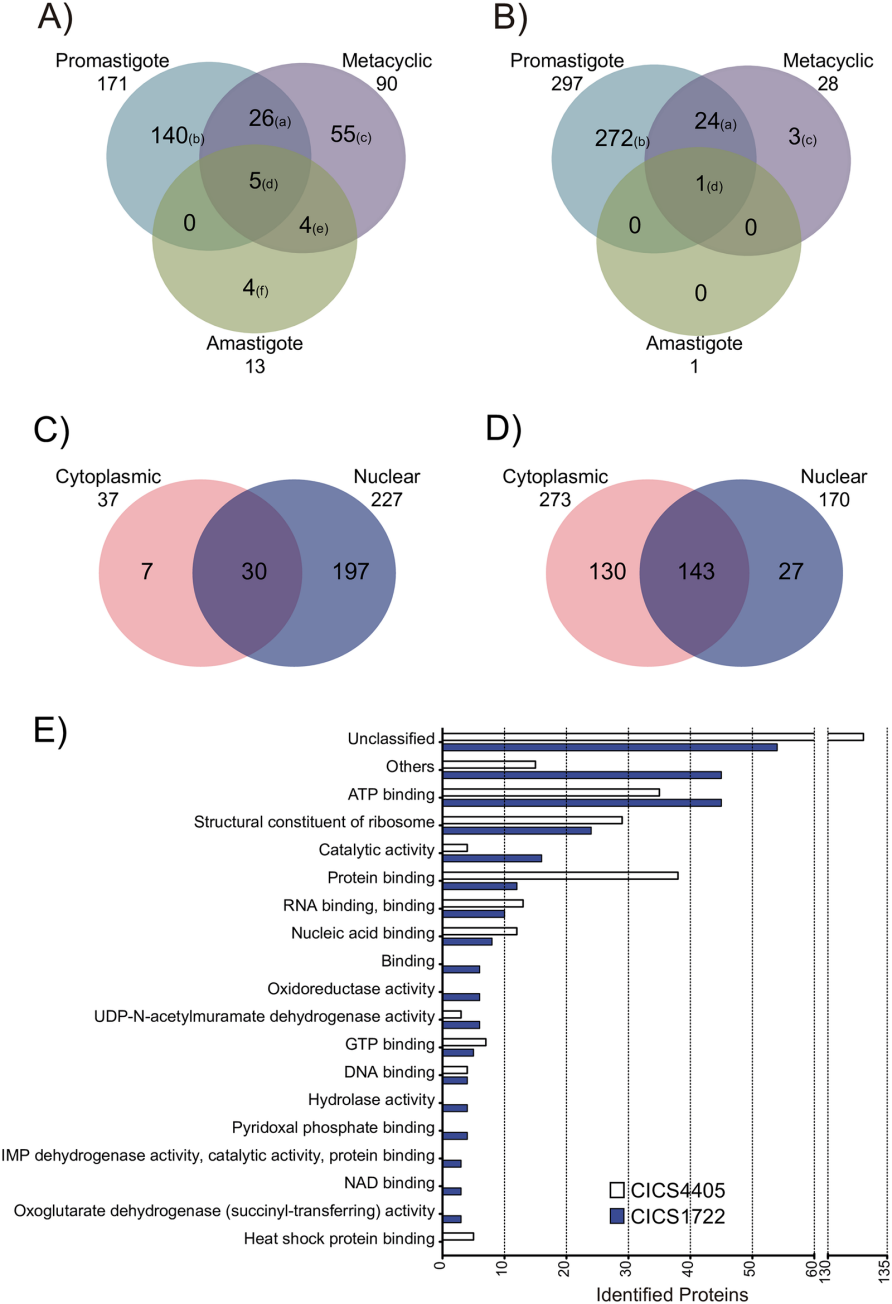
Proteins interacting with CICS during differentiation. The pull-down experiment was conducted using nuclear or cytoplasmic protein extracts of *L*. *donovani* in procyclic promastigotes (mid-log-phase), metacyclic promastigote cells (three days after the stationary phase) and axenic amastigotes. The resulting pull-downs were subjected to SDS-PAGE, and the band at the top of the gel, containing all of the proteins, was excised and submitted to mass spectrometry. The identified proteins were compared, and the common proteins were grouped in the Venn diagram according to differentiation stage for **(A)** CICS1722 and **(B)** CICS4405, or according to protein extract for **(C)** CICS1722 and **(D)** CICS4405. The proteins identified in each phase of differentiation are listed in the S3 Table according to identification letter. **(E)** Molecular function of the proteins associated with CICS1722 and CICS4405. Unclassified proteins are those without classification in Gene Ontology; those grouped as “Others” represent all Gene Ontology categories with less than three identified proteins in the analysis.

To determine if different proteins would interact with the same CICS throughout the life cycle, CICS/protein co-purifications were also assessed in metacyclic promastigote-enriched and amastigote cultures. Proteins extracted from the nucleus and cytoplasm were used in the pull-down assays. To identify the CICS binding proteins responsible for opposite expression profiles during differentiation, only CICS1722 and CICS4405 were selected for these pull-down experiments (Table 2 and Fig 1). To prevent loss of individual bands due to the low sensitivity of protein gel staining, proteins co-purified in pull-down experiments were not separated on SDS-PAGE but were recovered from a single band at the top of the running gel and sequenced by liquid chromatography-mass spectrometry (LC-MS). The proteins bound to the beads during the protein extract cleaning process were also sequenced and used as negative control. A total of 234 unique proteins co-purified with CICS1722 (Fig 4A, 4C and 4E—S1 Table) and 300 unique proteins co-purified with CICS4405 (Fig 4B, 4D and 4E—S2 Table) were retrieved from the nucleus and cytoplasm over the three stages of the parasite’s life cycle. For each analyzed CICS, we identified a group of stage-specific binding proteins (proteins that were bound to the CICS in different life cycle stages). Five proteins co-purified with CICS1722 were identified in all the life stages tested, three of which were nucleic acid binding proteins: LiAlba1 (LinJ.13.0270), LiAlba3 (LinJ.34.2410) and LinJ.35.2240 (RBP DRBD, putative). The co-purification of both CICS1722 and 4405 with the proteins LiAlba3 and RBP DRBD suggests that these proteins may be hub proteins, participating in the cores of different regulatory complexes.

A total of 140 proteins co-purified with CICS1722 only in the promastigote stage, 55 proteins only in the metacyclic stage, and 4 proteins only in the amastigote stage (Fig 4A). In contrast, only one protein was identified as co-purifying with CICS4405 in all the parasite stages: the RBP LinJ.35.2240. The same protein co-purified with CICS1722 in all stages. A total of 272 proteins from procyclic promastigotes and 3 proteins from the metacyclic stage were uniquely identified as co-purifying with CICS4405 (Fig 4B). Grouping of these proteins according to their subcellular localization shows a preference of CICS1722 for binding to nuclear proteins (Fig 4C), in contrast with CICS4405, which tends to bind cytoplasmic proteins (Fig 4D). Most proteins that co-purified with both CICS are binding proteins (ATP binding, RNA binding and nucleic acid binding—Fig 4E), reinforcing the hypothesis of CICS as protein-binding sites in mRNA.

**Fig 5:**
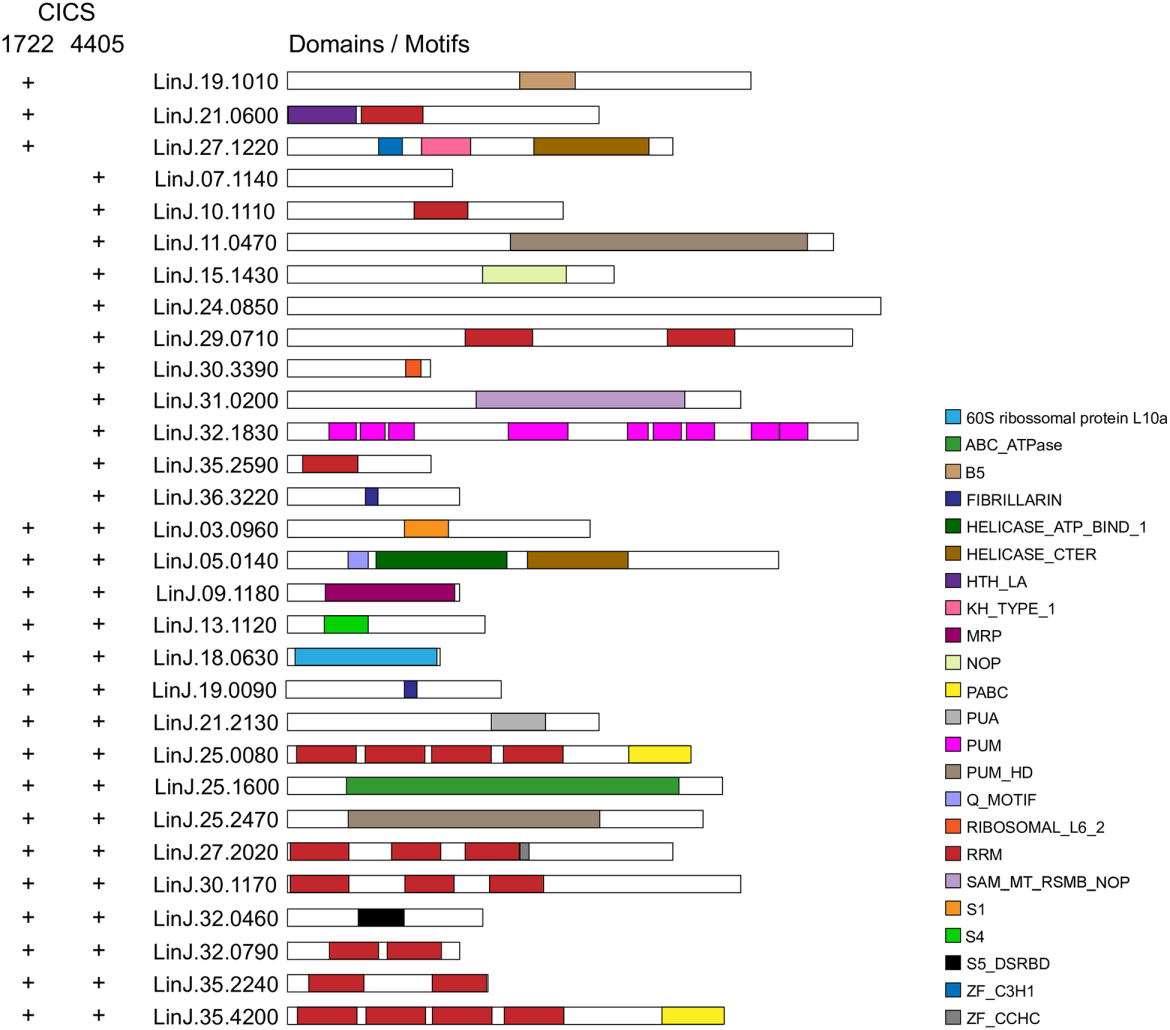
Domain distribution of *L*. *infantum* RBPs found to interact with CICS. The search was performed using Prosite (ExPASy). The interacting CICS is indicated by a plus (+) symbol on the right side of the protein. Different domains are represented by specific colors as shown in the key. Gene descriptions and categories are listed in S4 Table.

Identification of RBP motifs using Prosite / ExPASy [23] revealed a predominance of proteins containing the eukaryotic RNA recognition motif (RRM) (Fig 5 and S4 Table); nine proteins containing the RRM motif co-purified with CICS4405, and six of those also co-purified with CICS1722. A total of thirty proteins were classified according to the presence of 23 different motifs. From these only three (Lin.19.1010, a putative phenylalanyl-tRNA synthetase; LinJ.21.0600, a putative RBP; and LinJ.27.1220, a hypothetical conserved protein) were found solely co-purified with CICS1722 (Fig 5 and S4 Table).

In addition to those proteins found in both pull-down experiments with CICS1722 and 4405, ~46% of the proteins bound selectively to one or the other CICS. Because most known RBP domains have multiple functions, as shown in diverse model organisms, it is difficult to go beyond the identification of putative domains without functional correlations.

## 4 Discussion

The main purpose of this study was to elucidate part of the machinery involved in *Leishmania* gene expression control by revealing *cis*-acting elements and candidate *trans*-acting partner proteins. A computational investigation was conducted, searching for conserved sequences in the intercoding sequences of the known genomes of three different species: *L*. *braziliensis*, *L*. *infantum* and *L*. *major*. We assumed that sequence conservation in divergent regions could indicate selective pressure [11, 24]. It was hypothesized that the CICS might be involved in gene expression regulation, and a search was done for CICS that were part of the predicted 3' UTRs of groups of genes sharing similar patterns of expression. Similar expression profiles and a shared conserved *cis*-element in the 3' UTR could suggest that such groups of genes represent post-transcriptional regulons.

Among the examined CICS, the study verified CICS1722 as a *cis*-element acting in the regulation of gene expression. We demonstrated that CICS1722 had a negative effect on the modulation of mRNA stability and identified putative CICS1772-binding *trans*-regulatory proteins.

The removal of CICS1722 from the 3' UTR affected the control of NEO transcript levels in all life cycle stages of the parasite (Fig 2). There was a twofold increase in reporter transcript levels when CICS1722 was absent from the UTR. The observed variations in transcript levels could be due to increased mRNA stability. In fact, blocking both transcription and SLRNA methylation (via treatment with Sinefungin and Actinomycin D) led to increased levels of reporter transcript lacking CICS1722 in both procyclic promastigotes and amastigotes compared to reporter transcript bearing the intact 3' UTR, consistent with more stable transcripts in the absence of CICS1722 (Fig 3). Thus, CICS1722 may play a role in mRNA degradation, since the absence of the CICS resulted in an accumulation of reporter mRNA in the cell. Changes in reporter transcript levels were not observed in the absence of CICS4405, indicating that this effect is not common to all CICS and is not related to random changes in the 3' UTR.

The increased transcript stability in transcripts without CICS1722 may be due to changes in the secondary structure of the 3' UTR. RNA molecules tend to form highly stable secondary and tertiary structures *in vitro* and *in vivo* [25, 26]. These structures may be important for *trans*-acting factors to bind to mRNA, modify its stability, and participate in regulation of RNA-related cellular processes [27]. In fact, computational analysis of the secondary structure of the transcripts using the Vienna RNA website [28] revealed that CICS1722 removal led only to the loss of the loop containing CICS1722 (S5A and S5B Fig), with no changes to the rest of the structure. This suggests that the loop containing CICS1722 may play a central role in interaction with RBPs, resulting in destabilization of mRNA containing CICS1722. A similar analysis of CICS4405 indicates that its removal causes several changes in the region containing the CICS, including decreased size of the loop containing the CICS and the formation of internal loops. However, these changes do not seem to interfere with the role of the 3' UTR in this case (S5C and S5D Fig).

Herein it was demonstrated that CICS1722 in the 3' UTR environment enables binding to RBPs and negatively controls mRNA stability. Conversely, while CICS4405 also presents a binding site for RBPs, some of which also bind CICS1722, its removal did not affect mRNA stability. While it is reasonable to speculate that CICS4405 might be a *cis*-element involved in downstream events controlling gene expression, such as initiation of translation, at this point we have no data to support or refute this hypothesis, which inspires further investigation.

Since regulatory *cis*-elements provide binding sites for proteins involved in the control of gene expression, a search for CICS-associated RBPs was conducted using RNA pull-down experiments (Table 3 and Fig 4). Most of the peptides identified using this approach are nucleic acid binding proteins (Table 3 and Fig 4E). Interactions between proteins and nucleic acids (DNA or RNA) are vital for recognizing, maintaining and accessing genetic information [29, 30]. This interaction can regulate the transcription of nearby genes, not only for rapid response during stress but also for long-term adaptation in cell physiology [31, 32]. Both LiAlba3 and the RBP LinJ.35.2240 might act as hub proteins within these RNA-protein complexes involving the tested CICS, since these proteins were found to co-purify with all the CICS tested in different conditions (Table 3, Fig 4 and S3 Table). The RBP LinJ.35.2240 has two RRM domains. These domains constitute the most abundant RNA-binding domains in higher vertebrates. They are involved in post-transcriptional events of gene expression regulation, ranging from mRNA and rRNA processing to RNA transport, localization and stability [33, 34]. LiAlba3 contains an ALBA domain previously shown to interact with the translation machinery in *Trypanosoma brucei* [35] and with *cis*-elements involved in the control of stability of *amastin* transcripts in *Leishmania* [36]. Similar to the RBP LinJ.35.2240, LiAlba3 co-purified with all the tested CICS across different life cycle stages. Identification of these proteins as candidate partners of RNA *cis*-elements supports the hypothesis that CICS may regulate gene expression and represents a first step in the characterization of ribonucleoprotein complexes (RNPs) that participate in the control of gene expression.

Although pull-down experiments were conducted with the relevant negative controls, we cannot exclude possible promiscuity of binding between RBPs and short RNA sequences in an *in vitro* assay that would not occur *in vivo* in the parasites. Therefore, to confirm the interaction of these Alba and DRBD proteins *in vivo* with the studied CICS, *in vivo* crosslinking experiments followed by pull-down and MS analysis must be conducted.

In addition, stage- and CICS-specific binding proteins were also identified. The hypothetical protein LinJ.32.1000 was found to interact only with CICS1722 in procyclic promastigotes of the parasite. This protein has a thermonuclease domain, which is related to the hydrolysis of DNA and RNA at the 5' position of the phosphodiester bond, yielding 3'-mononucleotides and dinucleotides [37]. Interaction with a thermonuclease could explain the rapid degradation of mRNA containing CICS1722, a feature that was lost if the same 3' UTR was tested in the absence of CICS1722. It is worth noting that one of the proteins binding exclusively to CICS 4405 (encoded by LinJ.32.1830) was found to bear a PUM domain; this sequence motif is known to interact with the 3' untranslated region (3' UTR) of specific target mRNAs and repress their translation [38].

Interestingly, a common feature of pull-down experiments was a decrease in the number of proteins identified as interacting with tested RNAs as the life cycle progressed from procyclics to metacyclics to amastigotes. Whether this is due to a technical issue or represents a novel biological phenomenon must be further investigated. The interaction mechanism between CICS and proteins seems to be life-stage-specific. In the promastigote stage, 171 and 297 proteins were co-purified with CICS1722 and 4405, respectively. In the metacyclic stage, these numbers decreased to 90 and 28, and in the amastigote stage, only 13 proteins and 1 protein were found to be interacting with CICS1722 and 4405, respectively. Interestingly, the interactions with the Alba protein LiAlba3 and the RBP LinJ.35.2240 seem to be independent of both life cycle stage and CICS sequence.

When separately evaluating nuclear and cytoplasmic extracts for specific RNA-binding activity, it was worthy of note that most of the proteins binding CICS1722 came from different subcellular origins than those binding CICS4405. The number of nuclear proteins interacting with 1722 was 197, compared to 7 cytoplasmic proteins. This balance was inverted for CICS 4405: 27 nuclear and 130 cytoplasmic. Because we used the same extracts for both pull-down experiments, it is unlikely that the difference observed occurred due to a technical problem. An *in vivo* crosslinking experiment followed by subcellular fractionation and pull-down must be conducted to confirm this *in vitro* assay result and examine the potential biological significance of this finding.

Interestingly, the observed difference might be related to distinct mechanisms of gene expression control involving each of these *cis*-elements. Consistent with this, we found more nuclear proteins associated with *cis*-element 1722, which is part of a complex with a role in RNA stability. In contrast, the CICS4405-protein complex, rich in cytoplasmic proteins, may be involved in downstream processes controlling gene expression.

Here, we present results that suggest that CICS1722 is a site for protein binding and that it may be a *cis*-element within an RNP complex involved in the regulation of gene expression in *Leishmania donovani*. The current work indicates that the CICS databank may be a good resource for unraveling the machinery involved in gene expression regulation in *Leishmania*.

## Supporting information

S1 Fig Cell culture and *in vitro* differentiation conditions. Schematic representation of *in vitro* differentiation of *L*. *donovani* BOB promastigotes into axenic amastigotes and sample uptake. The axenic promastigote cultures were maintained at 26°C in M199, supplemented with 10% FCS. The green ellipse represents the promastigote stage (between the 4th and 5th day of culture); the purple ellipse represents the culture phase known as metacyclic (three days after the beginning of the stationary phase). The enriched metacyclic cultures (purple ellipse) were maintained in intermediary medium for 24 hours and then transferred to differentiation medium (time 0 h in the differentiation procedure).
(TIF)

S2 Fig Replacement of one allele of the endogenous gene by NEO, with or without CICS. (A) Scheme of the genomic region of the gene LinJ.31.1630, one of the genes bearing CICS1722 (purple bar) in the 3' UTR. The schematic represents: the original locus; the locus replaced by the reporter gene NEO, retaining the CICS (311630::NEO-1722); and the locus replaced by the reporter gene NEO lacking the CICS (311630::NEO-Δ1722). (**B**) Schematic representation of the genomic region of the gene LinJ.07.0150, one of the genes bearing CICS4405 (blue bar) in the 3' UTR. The scheme represents: the original locus; the locus replaced by the reporter gene NEO, retaining the CICS (070150::NEO-4405); and the locus replaced by the reporter gene NEO lacking the CICS (070150::NEO-Δ4405). The small black arrows in panels **A** and **B** indicate the annealing positions for the primers used in the PCR to confirm the correct integration of DHFR-NEO and NEO-DHFR, which anneal in the NEO gene and upstream within the recombined region. The N and S in panels **A** and **B** represent the restriction sites for *Nde*I and *Sma*I, respectively. The *Nde*I site was used to remove the CICS of the 3' UTR in the synthetic constructs. Open arrows represent the genes up- and downstream of the genes we used in our study. Confirmation of correct genomic integration was performed by PCR **(C** and **E)** and Southern blotting **(D** and **F)**. A fragment of ~300 bp of the NEO gene was used as a probe in the Southern blotting experiment with *Sma*I-digested genomic DNA of each transfectant. (**G** and **H)** Genomic DNA from transfectants was extracted and the region containing or not the CICS was amplified in the transfectants using primers annealing in the NEO and 3' UTR sequences (green triangles). The PCRs were sequenced and the lack of CICS (pink triangle) is represented by a gap in the consensus sequence (consisting of 4 sequencing replicates for each primer).
(TIF)

S3 Fig Localization and conservation of CICS in the 3' UTR of each gene bearing them. The position of the CICS (Query) within the 3' UTR of each gene (Subject) is depicted by a nucleotide number counting from the first nucleotide after the stop codon of the gene. Alignments were produced with BlastN^®^ using the CICS sequences listed in Table 2 and the *L*. *infantum* genome.
(TIF)

S4 Fig Proteins interacting with CICS. Pull-down was carried out using biotinylated RNA corresponding to each CICS and promastigote nuclear extract of *L*. *donovani*. The CICS used in the pull-down experiment are indicated at the top of the gel. The control (CTR) was a biotinylated RNA fragment of 28 nt not present in the *Leishmania* transcriptome. The gels were stained with Coomassie Blue and the excised bands (indicated in lowercase) were submitted to mass spectrometry (MS).
(TIF)

S5 Fig 3' UTR secondary structure in the presence and absence of CICS. Schematic representation of the predicted centroid secondary structure for the annotated 3' UTR of the gene LinJ.31.1630 with **(A)** or without **(B)** CICS1722 and the annotated 3' UTR of the gene LinJ.07.0150 with **(C)** or without **(D)** CICS4405. Predictions were performed by The Vienna RNA Website [28]. The colors show positional entropy according to the scale below each structure. The black arrow indicates the first base of the CICS in panels A and C. CICS are shown as white bases.
(TIF)

S1 Table Identified proteins interacting with CICS1722. Proteins from the promastigote (pro), metacyclic (met) and amastigote (ama) stages were divided into nuclear (nuc) and cytoplasmic extracts (cyto). For detailed legend, see sheet 0.
(XLSX)

S2 Table Identified proteins interacting with CICS4405. Proteins from the promastigote (pro), metacyclic (met) and amastigote (ama) stages were divided into nuclear (nuc) and cytoplasmic extracts (cyto). For detailed legend, see sheet 0.
(XLSX)

S3 Table Proteins interacting with both CICS1722 and CICS4405 in the nucleus and cytoplasm in each life stage of *L*. *donovani*. (XLSX)

S4 Table Motif identification for RBPs retrieved in RNA pull-downs. (XLSX)
