# Supplementary figures and images for: Disclosing 3' UTR cis-elements and putative partners involved in gene expression regulation in Leishmania spp
Source: PLoS One. 2017 Aug 31;12(8):e0183401. doi: 10.1371/journal.pone.0183401 (PMC5578504; doi:10.1371/journal.pone.0183401)

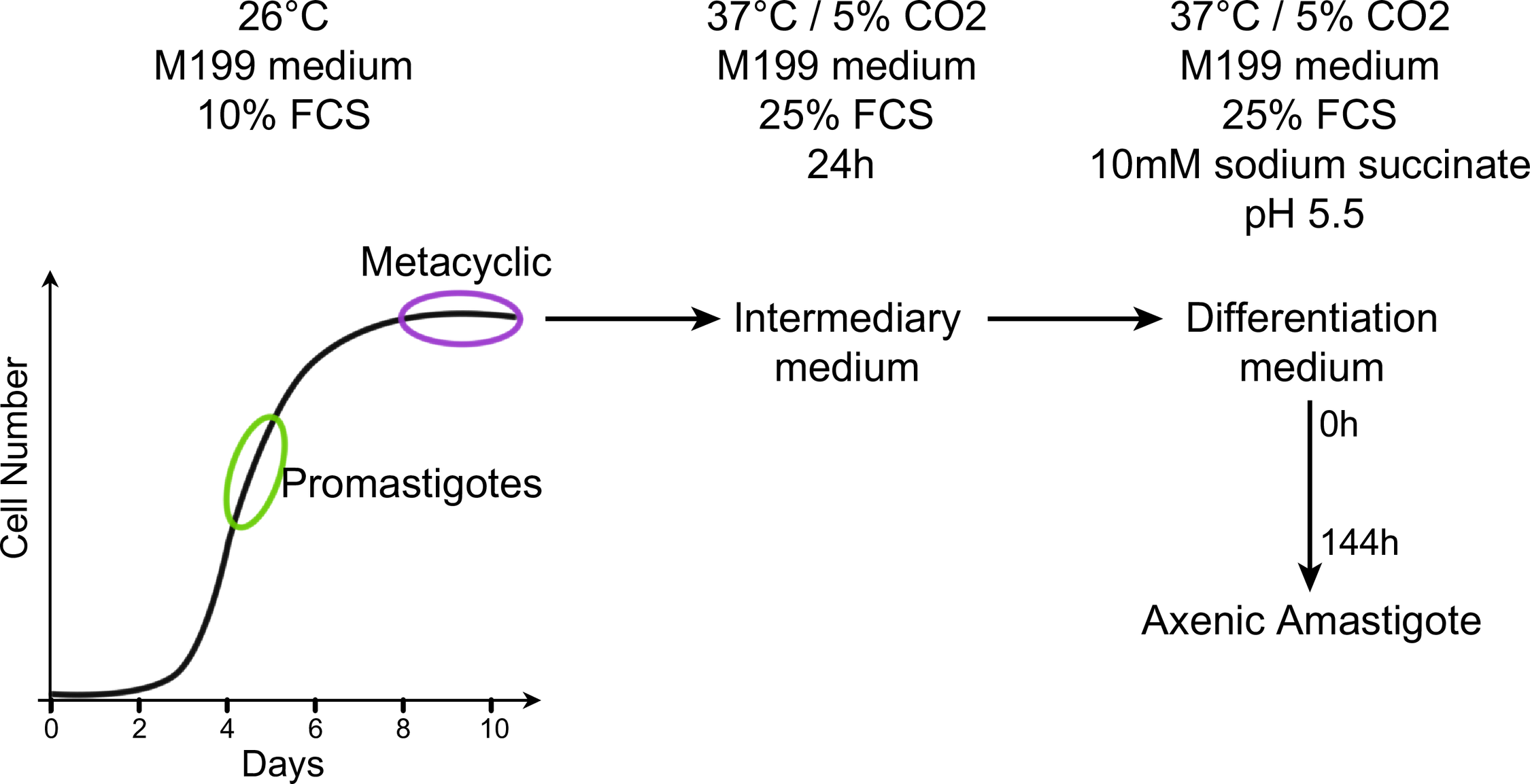

Supplement: S1 Fig — Schematic representation of in vitro differentiation of L. donovani BOB promastigotes into axenic amastigotes and sample uptake. The axenic promastigote cultures were maintained at 26°C in M199, supplemented with 10% FCS. The green ellipse represents the promastigote stage (between the 4th and 5th day of culture); the purple ellipse represents the culture phase known as metacyclic (three days after the beginning of the stationary phase). The enriched metacyclic cultures (purple ellipse) were maintained in intermediary medium for 24 hours and then transferred to differentiation medium (time 0 h in the differentiation procedure). (TIF) [file pone.0183401.s001.tif]

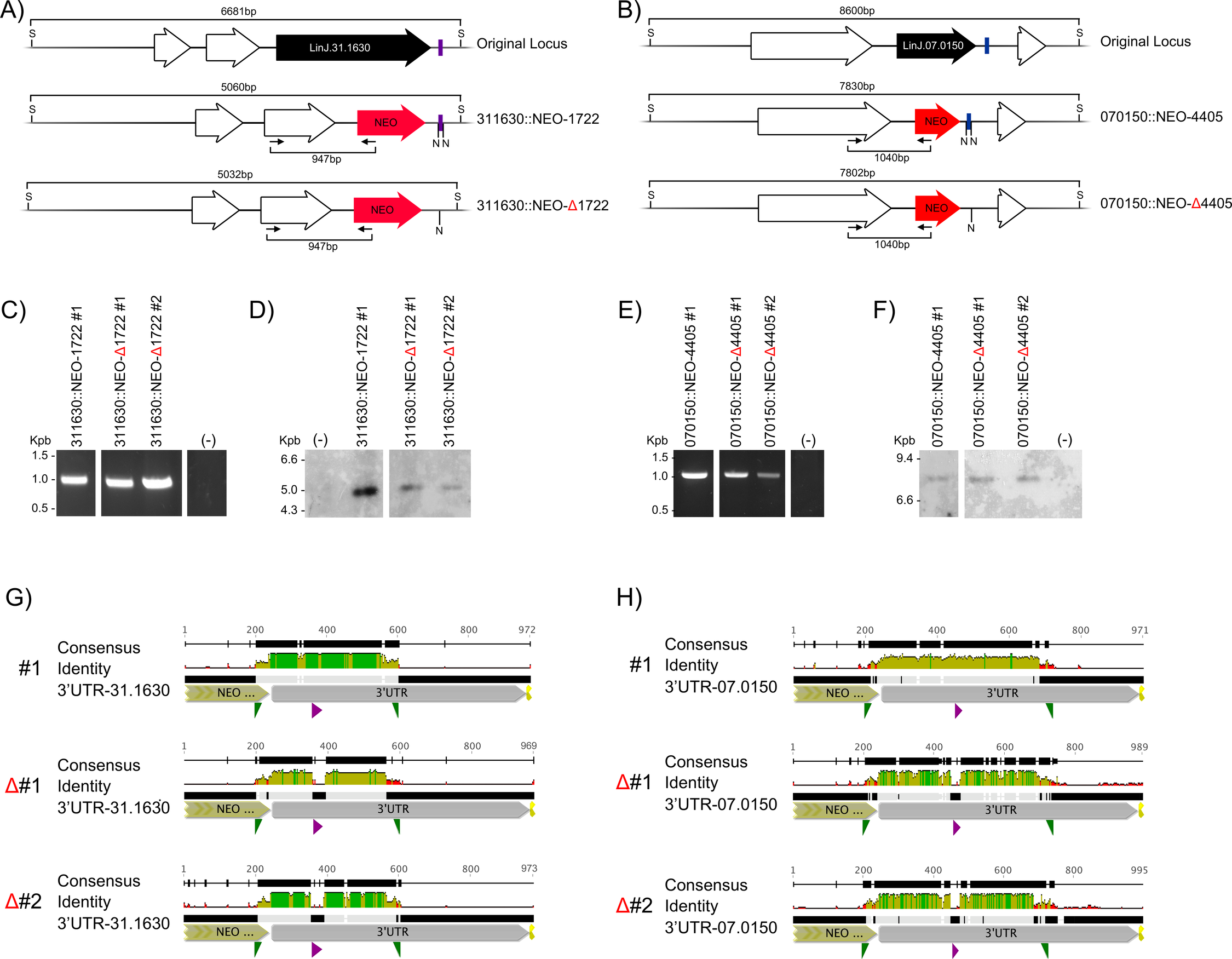

Supplement: S2 Fig — (A) Scheme of the genomic region of the gene LinJ.31.1630, one of the genes bearing CICS1722 (purple bar) in the 3' UTR. The schematic represents: the original locus; the locus replaced by the reporter gene NEO, retaining the CICS (311630::NEO-1722); and the locus replaced by the reporter gene NEO lacking the CICS (311630::NEO-Δ1722). (B) Schematic representation of the genomic region of the gene LinJ.07.0150, one of the genes bearing CICS4405 (blue bar) in the 3' UTR. The scheme represents: the original locus; the locus replaced by the reporter gene NEO, retaining the CICS (070150::NEO-4405); and the locus replaced by the reporter gene NEO lacking the CICS (070150::NEO-Δ4405). The small black arrows in panels A and B indicate the annealing positions for the primers used in the PCR to confirm the correct integration of DHFR-NEO and NEO-DHFR, which anneal in the NEO gene and upstream within the recombined region. The N and S in panels A and B represent the restriction sites for NdeI and SmaI, respectively. The NdeI site was used to remove the CICS of the 3' UTR in the synthetic constructs. Open arrows represent the genes up- and downstream of the genes we used in our study. Confirmation of correct genomic integration was performed by PCR (C and E) and Southern blotting (D and F). A fragment of ~300 bp of the NEO gene was used as a probe in the Southern blotting experiment with SmaI-digested genomic DNA of each transfectant. (G and H) Genomic DNA from transfectants was extracted and the region containing or not the CICS was amplified in the transfectants using primers annealing in the NEO and 3' UTR sequences (green triangles). The PCRs were sequenced and the lack of CICS (pink triangle) is represented by a gap in the consensus sequence (consisting of 4 sequencing replicates for each primer). (TIF) [file pone.0183401.s002.tif]

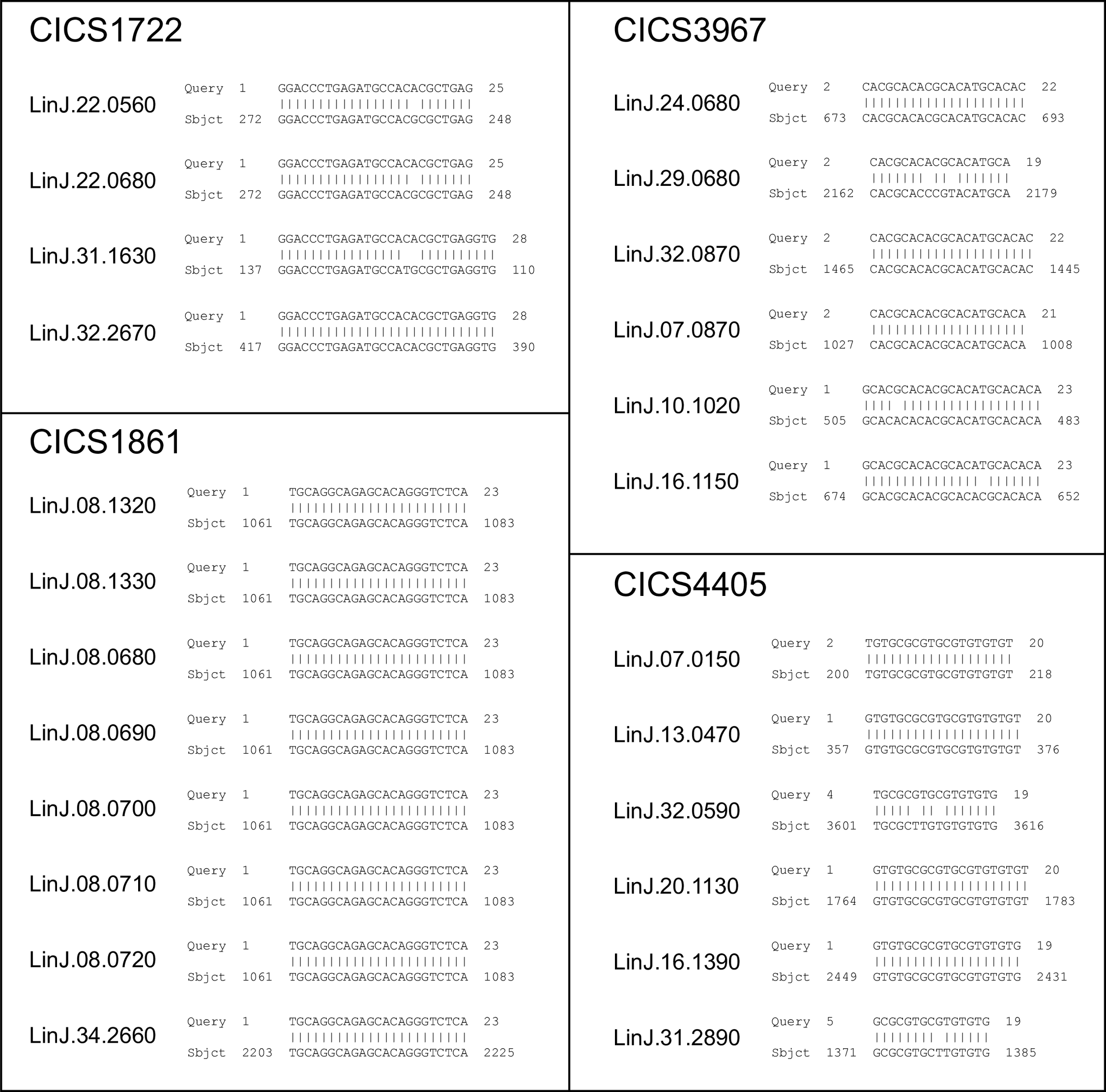

Supplement: S3 Fig — The position of the CICS (Query) within the 3' UTR of each gene (Subject) is depicted by a nucleotide number counting from the first nucleotide after the stop codon of the gene. Alignments were produced with BlastN® using the CICS sequences listed in Table 2 and the L. infantum genome. (TIF) [file pone.0183401.s003.tif]

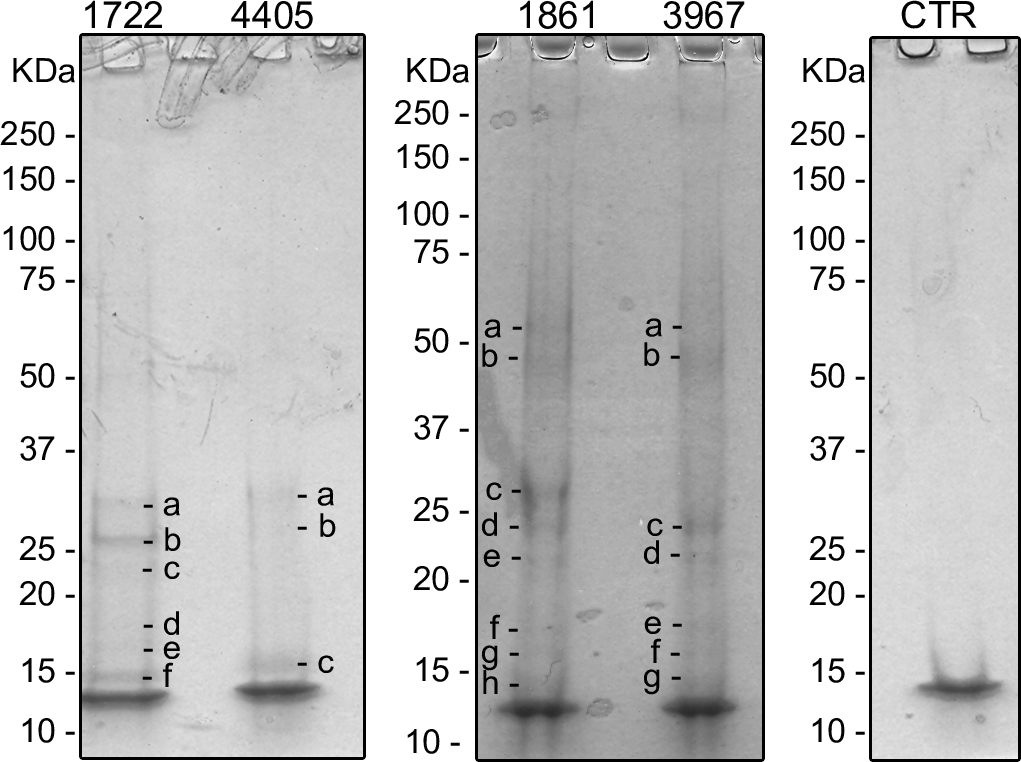

Supplement: S4 Fig — Pull-down was carried out using biotinylated RNA corresponding to each CICS and promastigote nuclear extract of L. donovani. The CICS used in the pull-down experiment are indicated at the top of the gel. The control (CTR) was a biotinylated RNA fragment of 28 nt not present in the Leishmania transcriptome. The gels were stained with Coomassie Blue and the excised bands (indicated in lowercase) were submitted to mass spectrometry (MS). (TIF) [file pone.0183401.s004.tif]

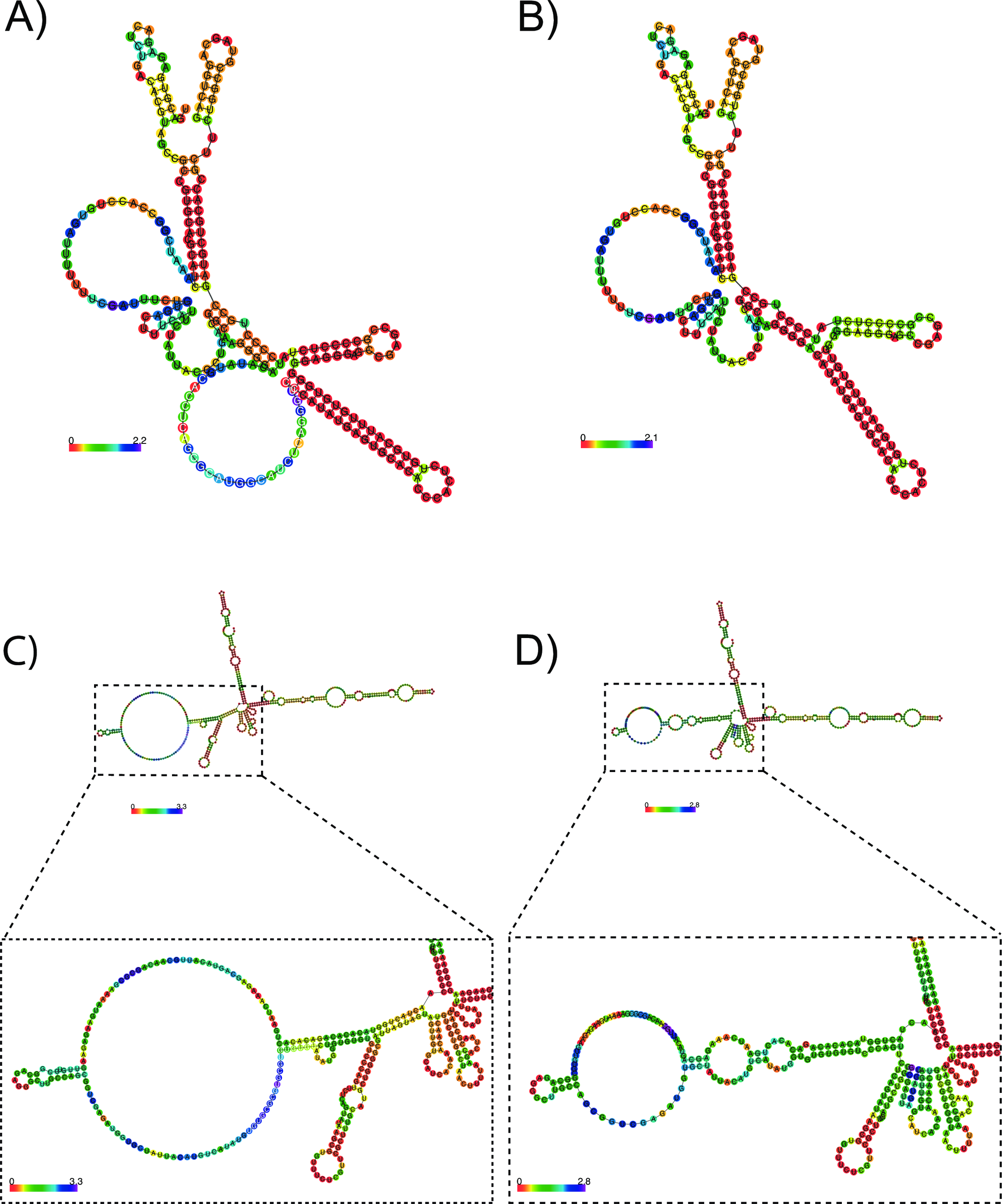

Supplement: S5 Fig — Schematic representation of the predicted centroid secondary structure for the annotated 3' UTR of the gene LinJ.31.1630 with (A) or without (B) CICS1722 and the annotated 3' UTR of the gene LinJ.07.0150 with (C) or without (D) CICS4405. Predictions were performed by The Vienna RNA Website [28]. The colors show positional entropy according to the scale below each structure. The black arrow indicates the first base of the CICS in panels A and C. CICS are shown as white bases. (TIF) [file pone.0183401.s005.tif]
